# The safety and feasibility of using a 5-Fr guiding catheter with a 0.035-inch guidewire in place for cerebral angiography

**DOI:** 10.1097/MD.0000000000036896

**Published:** 2024-01-05

**Authors:** Xianchen Huang, Guanqiang Li, Bo Hu, Xicheng Zhang, Yuan Sun

**Affiliations:** a Department of Vascular Surgery and Interventional Radiology, Dushu Lake Hospital affiliated to Soochow University, Suzhou, China.

**Keywords:** cerebral angiography, guided catheterization method, guidewire exchange, guiding catheter, polymer emboli

## Abstract

**Background:**

This study aimed to evaluate the safety and efficacy of performing diagnostic cerebral angiography using a 5-Fr guiding catheter with a 0.035-inch guidewire in place.

**Methods:**

Actual flow rates at different pressures using the 5-Fr guiding catheter with a 0.035-inch guidewire in place were measured in vitro. Integrity of the guidewire surface after high-pressure injection was determined by examination under a light microscope and scanning electron microscope. Injected and unused contrast medium were collected and analyzed using a particle detector. Furthermore, a prospective randomized controlled study was conducted to compare safety and efficacy between the guided (guidewire in place) and conventional methods.

**Results:**

The maximum injection pressure at a flow rate of 5 mL/s for the various types of commonly used contrast medium was approximately 350 psi, which is below the pressure limit for cerebral angiography. The guidewire surface remained relatively intact after multiple high-pressure injections. Procedure success and primary success rates did not significantly differ between the guided and conventional methods. However, procedure time (25.93 ± 4.07 vs 31.55 ± 5.49 minutes) and radiation exposure time (12.16 ± 3.82 vs 17.27 ± 6.12 minutes) were significantly shorter in the guided method group.

**Conclusion:**

The guided catheterization method is safe and feasible for cerebral angiography and has several advantages over the conventional method.

## 1. Introduction

Catheter-based angiography remains the gold standard for imaging of the cerebral vasculature.^[1]^ Use of a 0.035-inch hydrophilic guidewire advanced through a 4- or 5-Fr angiography catheter is common when performing cerebral angiography.^[2]^ Because the inner diameter of the catheter is only 0.038 inches, the 0.035-inch guidewire must be pulled out of the catheter for angiography. Alternatively, a Y valve can be used, which allows injection of the contrast medium (CM) through a 5-Fr guiding catheter (inner diameter, 0.058 inches) without removing the guidewire. Kwon et al called this technique the “guided catheterization method” and confirmed it was feasible to use for cerebral angiography after studying the effect of the guidewire on contrast injection pressure in vitro.^[3]^ Advantages of leaving the guidewire in place include easier navigation and improved stability of the catheter tip position. However, safety and efficacy have not been directly compared between the guided and conventional methods. This is important, as delamination of the hydrophilic coating on the guidewire after multiple high-pressure injections has the potential to induce significant adverse reactions.^[4–7]^

In this study, actual flow rates at different pressures were measured in vitro using the guided method. Integrity of the guidewire surface was also determined after multiple high-pressure injections. Furthermore, we conducted a prospective randomized controlled study to compare safety and efficacy between the guided and conventional methods in patients undergoing diagnostic cerebral angiography.

## 2. Methods

### 2.1. In vitro determination of the flow rate

A 0.035-inch guidewire (Terumo, Tokyo, Japan) was placed in a 5-Fr guiding catheter (Launcher Jr 3.5; Medtronic, Minneapolis, MN) and a Y valve was used to connect the guiding catheter and high-pressure syringe. The tip of the catheter was placed in an arterial silica gel model, which was connected to a peristaltic pump with the pressure varying between 90 and 140 mm Hg. The high-pressure syringe was set at different pressures and actual flow rates of various types of CM were measured.

### 2.2. Assessing the integrity of the hydrophilic guide wire after high-pressure injections

A 5-Fr guiding catheter with a 0.035-inch guidewire inside was connected to a high-pressure syringe as previously described.^[3]^ Iohexol (Jiangsu Hengrui Pharmaceutical Co., Ltd., Lianyungang, China) was injected 20 times at a pressure of 1000 psi and flow rate of 6 mL/s; injection volume was 10 mL. Both injected and unused CM were collected and examined for guidewire coating particles using an LE100 intelligent particle detector (Suzhou Sujing Group Co., Ltd., Suzhou, China). Particle number and size were recorded. In addition, the surface of the guidewire was examined under a light microscope and scanning electron microscope to evaluate the integrity of the guidewire.

### 2.3. Prospective randomized controlled study design

Patients referred for diagnostic cerebral angiography at Dushu Lake Hospital Affiliated to Soochow University were enrolled from June 2019 to December 2022. The study was approved by the ethics committees of Dushu Lake Hospital Affiliated to Soochow University. All patients provided written informed consent. Sample size was predicted using SPSS software version 28.0 (IBM Corp., Armonk, NY). Two hundred forty patients were randomly assigned to the guided (n = 120) and conventional angiography groups (n = 120). Exclusion criteria included age <18 or >80 years, history of CM allergy, renal insufficiency, and bilateral iliac artery occlusion.

Operation time, radiation exposure time, procedure success, and complications were recorded. Successful angiography performed using the initially selected catheter was defined as primary success. Complications were categorized as major or general. Major complications included angiography-related cerebrovascular accident. General complications included puncture site complications such as subcutaneous congestion, puncture site hematoma, pseudoaneurysm, and lower limb ischemia.

### 2.4. Procedural protocol

Cerebral angiography procedures were performed by a same team using right or left common femoral artery access. A 5-Fr pigtail catheter (Terumo) was used to perform aortic arch angiography to evaluate the aortic arch. In the guided method group, a 5-Fr guiding catheter (Launcher Jr 3.5; Medtronic) with 0.035-inch hydrophilic guidewire (Terumo) in place was connected to a flushing system and a syringe using a Y valve for selective catheterization. CM was injected through the syringe with the guidewire kept in place. After catheterization, the guidewire was returned to the tip of guiding catheter unless the catheter tip was not stable. If the vertebral artery was difficult to catheterize, the guidewire was cannulated into the vertebral artery and the guiding catheter tip was placed near the orifice of vertebral artery for angiography. Before high pressure injection, the Y valve was tightened to prevent the guidewire from migrating during injection. In the conventional group, a 4-Fr vertebral catheter (VER 135; Cordis, Miami Lakes, FL) with a preloaded wire was used for selective catheterization and cerebral angiography. During contrast injection, the guidewire was removed from the catheter. If angiography could not be successfully completed using the preferred catheter, other catheters (Hunterhead catheter or Siommons catheter; Cordis) were selected in both groups.

Iohexol (Jiangsu Hengrui Pharmaceutical Co., Ltd., Lianyungang, China) was used as CM and injected using the following parameters in both groups: flow rate, 4 mL/s; total volume, 8 mL; pressure, 300 psi. After the procedure, femoral pressure and a compression dressing were applied for hemostasis.

### 2.5. Statistical methods

Statistical analyses were performed using SPSS software version 28.0 (IBM Corp.). Continuous data was compared using the *t* test; Bonferroni's correction was applied when multiple paired comparisons were performed. Categorical variables were compared using the chi-square test or Fisher exact test. *P* < .05 was considered significant.

## 3. Results

### 3.1. Actual flow rate/volume of CM at different injection pressures

**Figure 1.**
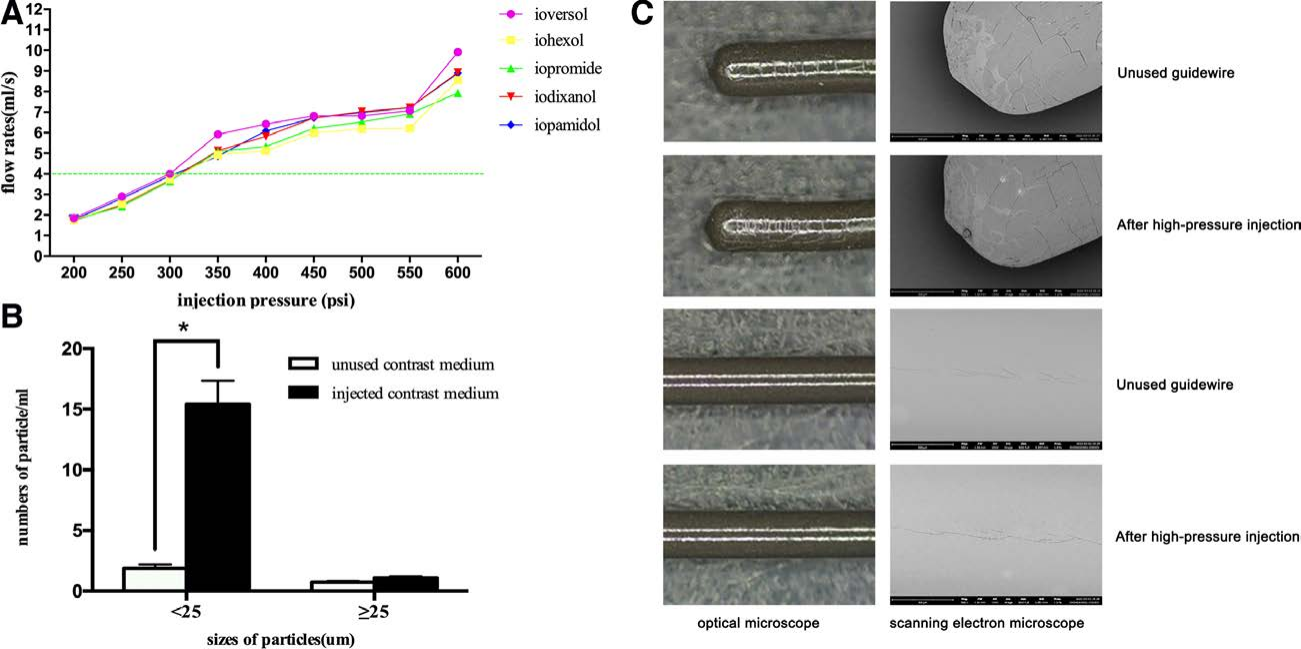
(A) The different type of CM achieved the target flow rate at different injection pressure. All of the CM could achieve the flow rate of 4 mL/s at approximately 300 psi. (B) The number of particles <25 µm in size was significantly higher in the injected CM than the unused medium, however, there was no significant difference of the number of particles ≥25 µm in size. (C) After the high-pressure injections, the entire outer surface of the guidewire was relatively smooth and uniform under the light microscope and scanning electron microscope. CM = contrast medium. *means *P* < 0.05.

The flow rate increased in conjunction with injection pressure for each type of CM (Fig. 1A). The target flow rate of 4 mL/s was achieved at approximately 300 psi for all types. At a flow rate of 5 mL/s, injection pressure varied significantly because of differing viscosities of the types of medium. The maximum actual pressure was approximately 350 psi, which is lower than the pressure limit for performing angiography.

### 3.2. Hydrophilic guidewire integrity assessment

The number of particles <25 µm in size was significantly higher in the injected CM than the unused medium. However, the number of particles ≥25 µm in size did not differ (Fig. 1B). According to “Pharmacopoeia of the People’s Republic of China,” intravenous injection with a labeled volume of 100 mL or more contain no more than 25 particles of 10 μm or more per 1 mL, and no more than 3 particles of 25 μm or more. The particle numbers in this study conformed to the national standard.

After the high-pressure injections, the entire outer surface of the guidewire was relatively smooth and uniform under the light microscope. Only a small number of scratches were seen on the tip of the guidewire, which was very similar in appearance to the surface of an unused guidewire. Scanning electron microscope showed several flakes of coating falling off and a number of cracks at the tip of the guidewire (Fig. 1C).

### 3.3. Clinical outcomes

Patient characteristics were similar between the guided and conventional angiography groups (Table 1). Angiography was successful in 114 patients (95.0 %) in the guided group and 116 patients (96.7%) in the conventional group (*P* = .517). The primary success rates were 77.5% and 75.8%, respectively (*P* = .760). However, procedure time (25.93 ± 4.07 vs 31.55 ± 5.49 minutes) and radiation exposure time (12.16 ± 3.82 vs 17.27 ± 6.12 minutes) were significantly shorter in the guided angiography group.

**Table 1:** Patient characteristics.

	Guided group (n = 120)	Conventional group (n = 120)	Inspection value	*P* value
Sex (F/M)	75/45	72/48	0.158	.691
Age (yr)	77.65 ± 7.16	77.37 ± 6.41	0.323	.747
Basic disease				
Hypertension	78	75	0.162	.687
Diabetes mellitus	54	59	0.418	.518
Coronary disease	32	38	0.726	.394
Hyperlipidemia	57	64	0.817	.366
Cerebral infarction	28	33	0.550	.459
Smoking	27	26	0.024	.876
Antiplatelet medication				
Single antiplatelet drugs	68	65	0.152	.697
Dual antiplatelet drugs	13	16	0.353	.552
Anticoagulant therapy	3	1	1.063	.302
Aortic arch type				
I	39	34	−0.635	.526
II	41	43		
III	40	84		

No procedure-related cerebrovascular accidents (infarction or bleeding) were observed. Incidence of minor complications was similar between the groups. One patient in the guided angiography group developed symptoms of lower limb ischemia after the procedure, which were relieved after balloon dilatation at the puncture site. Clinical outcomes are presented in Table 2.

**Table 2:** Clinical outcomes.

	Guided group (n = 120)	Conventional group (n = 120)	Inspection value	*P* value
Total success	114(95.0%)	116(96.7%)	0.420	.517
Primary success	93(77.5%)	91(75.8%)	0.093	.760
Operation time (min)	25.93 ± 4.07	31.55 ± 5.49	−9.004	˂.001
Radiation exposure time (min)	12.16 ± 3.82	17.27 ± 6.12	−7.752	˂.001
Cerebrovascular accident	0	0	-	-
Ecchymosis	12	15	-	-
Hematoma	6	4	-	-
Lower limb ischemia	1	0	-	-

## 4. Discussion

Compared with the conventional angiography method, guided catheterization with the guidewire in place has several advantages. First, CM can be injected at any time during the procedure without removing the guidewire to confirm target vessel and catheter location, which decreases procedural time and radiation exposure. Second, the guidewire remaining in place can provide support for the catheter, which can prevent it from falling out of the target vessel. In addition, the presence of the guidewire decreases the risk of air entering the catheter during guidewire exchange.

The shape of the 5-Fr Launcher Jr 3.5 guiding catheter resembles that of the Cobra catheter. However, the Launcher Jr can be manually molded into various shapes and it resists deformation after molding. Therefore, it can replace the vertebral catheter. In addition, its internal diameter is larger than the angiography catheter, which makes it possible to perform angiography with the guide wire in place. Furthermore, contrast imaging quality is comparable between the guided and conventional methods. Because the catheter and guidewire used in our study differed from those examined by Kwon et al,^[3]^ we determined actual flow rates of commonly used types of CM at different injection pressures. Our results confirmed that the 5-Fr guiding catheter with a 0.035-inch hydrophilic guidewire in place was able to provide the flow rate required for cerebral angiography (4–6 mL/s) with injection pressures less than 350 psi.

Embolism of hydrophilic polymer on the surface of the guidewire, which can result in cerebral vasculitis or vasculopathy, is a potential complication of leaving the guidewire in place during high-pressure injection.^[5,7]^ In our study, the surface of the guidewire maintained its integrity when examined after injection and relatively few particles of coating polymer were observed in the injected CM. However, the number of particles <25 µm in size was significantly higher in the injected medium than the unused medium. The consequences of this and the association of small particles with complications warrants further study, as small polymer emboli from vascular device surfaces may have unanticipated biological reactions.

**Figure 2.**
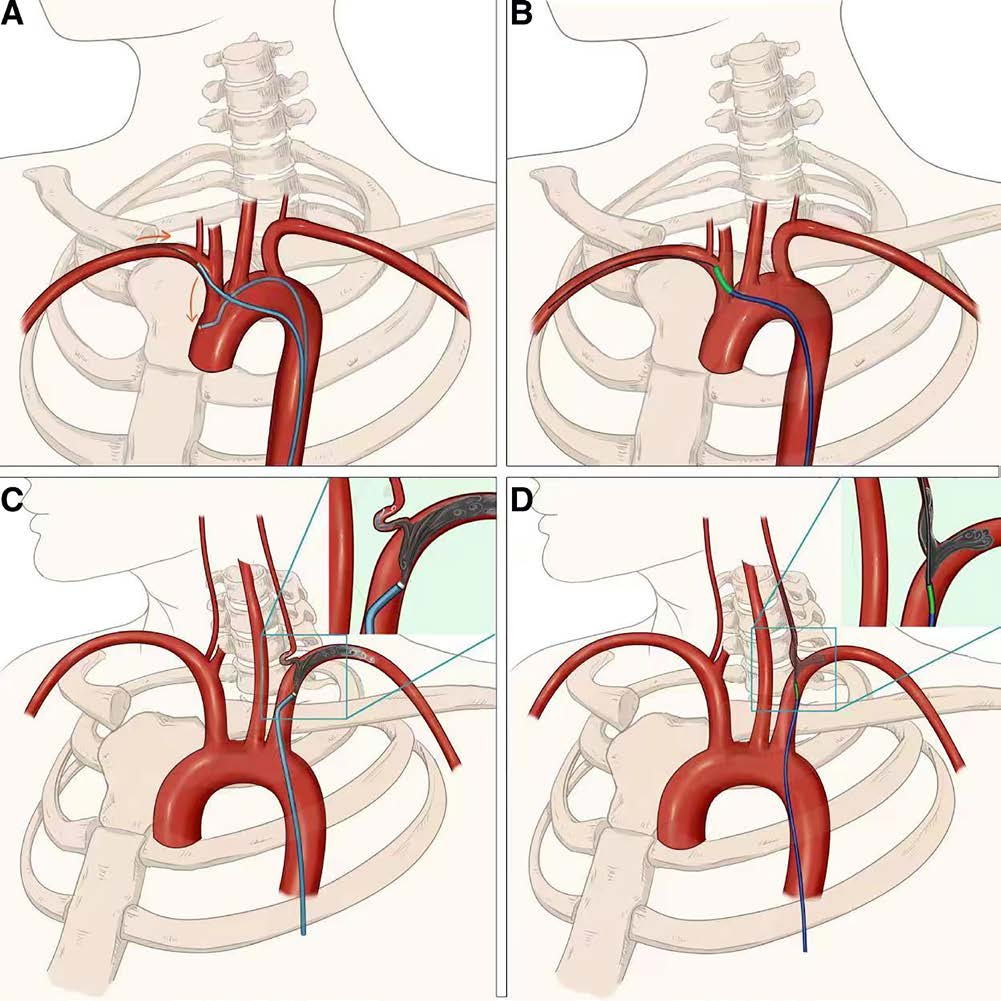
(A, B) During right vertebral arteriography, a guidewire in the distal end of the subclavian or axillary artery can increase catheter stability and prevent uncontrolled catheter movements. (C, D) For the distorted vertebral artery, most of the CM will flow into the subclavian artery if the catheter is placed in the subclavian artery. Keeping the guidewire in the vertebral artery through the guiding catheter can maintain the catheter at an appropriate angle, which can significantly improve imaging quality.

When performing angiography, the guidewire can be returned to the catheter to avoid interfering with angiography. Tortuosity of the aortic arch and branch vessels frequently cause catheter stability issues, which can be ameliorated by leaving the guidewire in place. This can increase catheter stability and prevent uncontrolled catheter movements that increase the risk of vasospasm, vessel damage, and plaque dislodgment. During right vertebral arteriography, the distance from the right subclavian artery origin to the orifice of the right vertebral artery is very short and maintaining the catheter in this arterial segment for contrast injection is difficult. However, with a guidewire in the distal end of the subclavian or axillary artery, maintain the guiding catheter in this segment is easier (Fig. 2A and B).

Sometimes, the catheter cannot directly enter the vertebral artery owing to distortion, so the catheter is placed in the subclavian artery. Considering the angle of the vertebral artery, most of the CM will flow into the subclavian artery, which may affect imaging quality. Keeping the guidewire in the vertebral artery through the guiding catheter can maintain the catheter at an appropriate angle, which can significantly improve imaging quality (Fig. 2C and D).

## 5. Conclusions

The guided catheterization method is safe and technically feasible for cerebral angiography. This method can decrease procedure time and provide stabilizing support for the catheter.

## Author contributions

**Writing – original draft:** Xianchen Huang.

**Methodology:** Guanqiang Li, Bo Hu.

**Data curation:** Bo Hu.

**Supervision:** Xicheng Zhang.

**Conceptualization:** Yuan Sun.

**Writing – review and editing:** Yuan Sun.
